# Affective Consequences of Social Comparisons by Women With Breast Cancer: An Experiment

**DOI:** 10.3389/fpsyg.2020.01234

**Published:** 2020-06-11

**Authors:** Katja Corcoran, Gayannee Kedia, Rifeta Illemann, Helga Innerhofer

**Affiliations:** ^1^Sozialpsychologie, Psychology Institute, Karl-Franzens University of Graz, Graz, Austria; ^2^Biotechmed, Graz, Austria

**Keywords:** social comparison, breast cancer, contrast, identification, self-esteem, self-efficacy, mood, depression

## Abstract

**Objective:**

People with severe illness often meet and compare themselves with other patients. Some of these comparison standards do well, others do poorly. Such comparisons could have positive as well as negative consequences depending on whether people identify or contrast from the standard. In the present study, we examine whether patients with breast cancer can benefit from comparisons by engaging in favorable comparison processes.

**Design:**

102 women diagnosed with breast cancer were randomly assigned to read a (fictitious) self-report from a well or poorly adjusted breast cancer patient.

**Main Outcome Measures:**

Participants reported their affective reaction (mood, anxiety, depression) and specified their comparison process (identification or contrast).

**Results:**

In general, participants engaged in favorable comparison processes by contrasting predominantly with poorly adjusted patients, and identifying with well-adjusted ones.

**Participants’ Mood Assimilated to the Standard:**

Participants reported more positive mood after having been exposed to the well-adjusted than the poorly adjusted standard.

**Anxiety and Depression Varied With the Type of Comparison Process:**

It was lower the more they avoided unfavorable comparisons (contrasting with the well-adjusted patient and identifying with the poorly adjusted one).

**Conclusion:**

Patients adjust their comparison processes to the standard to experience favorable comparisons. Especially avoiding unfavorable comparison processes reduces the risk of negative consequences after encountering other patients. Thus, patients may profit from comparisons as long as they engage in the right process.

## Introduction

Breast cancer is the most common form of cancer among women worldwide (Ferlay et al., 2019). In addition to the physical threats and pains, women with breast cancer face major psychological challenges. Breast cancer creates negative mood, anxiety, depression, and affects patients’ self-image (Soo and Sherman, 2015). While coping with this life-changing event, breast cancer patients are often exposed to the stories of other patients. They learn these stories in books, websites, and internet forums, as well as in the hospital during treatment or at self-help group meetings (Weis, 2003; Cipolletta et al., 2019). It is often argued that learning about the fate, difficulties, and resources of other patients is beneficial for people with serious illness. It would help them assess their situation, feel less alone, and find inspiration to overcome the challenges posed by their condition. However, research shows that comparisons with other people do not only have positive effects. They can also be threatening or discouraging when, for example, the person with whom one identifies is not doing well (see Ussher et al., 2006 for reports of patients attending self-groups) or when the comparison process leads to feeling less fortunate or weaker than others. The effects of social comparisons in patients facing serious illness, such as breast cancer, are thus so far unclear. This research aims to shed light on this issue.

Several studies have examined social comparisons among people with severe chronic conditions (Arigo et al., 2014). Much of this research has been conducted using a narrative method in which patients report natural comparisons and their subsequent reactions (Wood et al., 1985; Bogart and Helgeson, 2000; Cabrera-Perona et al., 2017). Overall, in these observational studies, patients primarily report a positive effect after a comparison, suggesting that social comparisons are an adaptive strategy for patients to maintain positive mood and cope with illness (Arigo et al., 2014). However, these studies should be interpreted with caution as they are based on spontaneous patient reporting and, therefore, are likely to be subject to several biases (e.g., patients may filter out negative experiences). Experimental research offers more controlled conditions for studying this issue.

Only a few experimental studies have been run to investigate the effects of social comparison in patients with severe disease. Their results are equivocal. For example, Stanton et al. (1999) asked patients with breast cancer to listen to the interview of another patient. The patients interviewed in the audiotapes varied concerning prognosis (good, poor, unspecified) and psychological adjustment (good: patient expressing positive emotions; poor: patient expressing high levels of distress; unspecified). Results indicated that women who had listened to the poorly adjusted standard reported feeling better about their own adjustment than those who have listened to the other standards. However, they also reported a more negative affect after listening to the audiotape than before, regardless of the standard. In another study (Van der Zee et al., 1998), breast cancer patients read about an upward (positive adjustment and prognosis) or downward (negative adjustment and prognosis) standard before assessing their affect and indicating their identification with that patient. In this study, in contrast to the results of Stanton et al., participants reported more positive affect after reading about the upward standard and this effect was qualified by the degree of identification: The more participants identified with the upward standard, the more positive their affect was. Thus, although patients with severe illness often report positive outcomes from social comparisons, the experimental evidence is less conclusive. Sometimes they seem to benefit from a comparison with a well-adjusted patient and sometimes from a comparison with a poorly adjusted patient. An explanation for this apparent contradiction may be found in social comparison theory.

Buunk and Ybema (1997) distinguish two comparison processes: identification and contrast. People identify with another person when they feel similar to that person and see her fate as a possible future for them. They then assimilate their feelings and self-evaluation to hers. Conversely, people contrast away from a comparison standard when they perceive him or her as different and are reminded of the fact that they themselves are doing better or worse (Gerber et al., 2018). The Selective Accessibility Model from Mussweiler (2003) focuses in more detail on the cognitive processes behind the two potential outcomes. Feeling similar or different from the other person again plays a key role. Mussweiler (2003) suggests that feeling similar to the comparison standard triggers a search for similarities on specific dimensions of comparison and makes this knowledge accessible. Since self-evaluation is based on accessible knowledge, this selective search leads to assimilation. Conversely, feeling different from the standard triggers a search for dissimilarities, which leads to contrast. Thus, both theoretical models posit that the consequences of a comparison depend not only on the person to whom one compares oneself, or on whether this person experiences positive or negative outcomes, but also on the type of comparison process (identification or contrast) one applies. Social comparison is a highly flexible process.

Patients with breast cancer may take advantage of the flexibility of social comparisons. Indeed, research in social psychology has shown that people facing threatening experiences use social comparisons to improve their self-image and feel better about their situation (for a review, see Wills, 1981). Hakmiller (1966) demonstrated this hypothesis experimentally. He gave subjects who had taken a personality test the threatening feedback that they had a high level of hostility toward their parents (vs. a low level of hostility in the control group). Subjects who received the threatening feedback showed an exaggerated tendency to compare themselves to someone who had received an even more hostile feedback. Subsequent studies have replicated this result and have shown that, given the opportunity to choose their standard of comparison, people under threat compare themselves to downward standards. Cancer patients also show this pattern: When they are interviewed, the spontaneous social comparisons they make are mainly with other patients who are worse off (Wood et al., 1985). But how do cancer patients react if they do not have the possibility to choose their comparison standard?

Previous scholars have assumed that cancer patients who cannot avoid the comparison with another patient would still be able to engage in favorable comparison processes by identifying with upward standards and contrasting with downward standards (Taylor and Lobel, 1989). Similarity appears to play a key role in inducing contrast or identification. However, similarity between two people is not a fixed or given fact, but rather highly subjective. People belong to multiple categories and can be characterized on many dimensions. Perceived similarity thus depends on the characteristics on which one decides to focus. When one encounters a person with whom one could compare oneself, it is thus possible to switch from a focus on similarities to a focus on dissimilarities, and thus from identification to contrast (Mussweiler et al., 2000). The question is whether people with a severe illness also use this strategy. If this were true, learning the story of any patient could lift their mood, decrease depression, and reduce anxiety. To our knowledge, this has not been experimentally tested yet.

The current project is designed to test the hypothesis that breast cancer patients flexibly adapt their comparison process (identification vs. contrast) to the standard (upward or downward) they are exposed to in order to promote positive outcomes. However, we do not expect this effect to be the same in all patients.

Previous research suggests that the tendency to engage in favorable comparisons depends on personality factors. Some people protect their positive self-concept better than others (Alicke and Sedikides, 2011). One personality factor related to motivated cognition is trait self-esteem. People with high self-esteem are more prone to adopt self-enhancing strategies than people with low self-esteem. They tend to overlook negative information about themselves (van Dellen et al., 2010), they are more likely to make self-serving attributions (e.g., Miller and Ross, 1975), to engage in compensatory self-enhancement after receiving negative feedback (e.g., Baumeister, 1982), and to derogate sources of negative feedback (e.g., Baumgardner et al., 1989). People with high self-esteem also tend to have high self-efficacy (Lane et al., 2004). Self-efficacy is the subjective feeling of being in control (Bandura, 1977). People with high self-efficacy often cope well with threatening situations (Folkman, 1984) and cultivate their feeling of empowerment by making self-serving attributions (Watt and Martin, 1994). Thus, patients with a high self-esteem and a high self-efficacy are likely to cope better with other patients’ stories by engaging in favorable comparison processes (Taylor and Stanton, 2007).

In the study presented in this article, we expected women with breast cancer to make favorable comparisons with other patients and we predicted that the more they did, the more beneficial the comparison would be for them. In other words, the more patient contrast with poorly adjusted standards and the more they identify with well-adjusted standards, the better they should feel, and the less anxiety and depression they should experience. In addition, we predicted that the higher women’s self-esteem and self-efficacy, the more positive comparisons they make and the greater the benefits they derive.

## Materials and Methods

In this study, women with breast cancer diagnosis were asked to read a self-report supposedly written by another patient. This self-report was actually the manipulation of the comparison standard. The self-report patient described either the difficulties and struggles she experienced in relation to her illness, depicting a rather depressive and hopeless picture (poorly adjusted standard), or she talked about the ease and speed of adjustment, even pointing out positive consequences from her experience (well-adjusted standard). Before and immediately after reading the self-report, participants indicated their mood. In addition, after reading the self-report, we assessed their feelings of depression and anxiety, the extent to which they identified or contrasted with the standard as well as their propensity to focus on similarities or differences. Prior to reading the self-report, we also measured participants’ self-esteem and self-efficacy.

### Participants

102 women with a breast cancer diagnosis participated in this study^1^. On average, the women were 63 years old (range 38–83) and 80% of them had received their first diagnosis more than two years ago (up to 33 years ago); 97 women had had at least one breast operation; 25 were currently in therapy, and 70 were attending a self-help group at the time of the study or had attended one in the past; 69 were married or living with a partner; 28 were currently working whereas 63 were retired (for a more detailed description of the sample, see Supplementary Table S1); 52 women read the self-report of the poorly adjusted patient and 50 the self-report of the well-adjusted patient.

### Procedure

To recruit participants, we contacted self-help groups in Styria, Austria, and the Styrian Cancer Society, as well as oncology stations at two hospitals and several centers for mammography in Graz, Austria. Women with breast cancer were made aware of the study by flyers and posters and by word-of-mouth recommendation. To conduct the study, a female investigator either met with the women individually or administered the questionnaire to a group of women during a self-help meeting. The women received a package of organic body products as a token of our gratitude and could participate in a small raffle.

The whole study was conducted as a paper and pencil study and was approved by the Ethics Commission of the University of Graz (Austria). Participants first read a short information sheet about the study, they gave their informed consent to participate, and then filled out the questionnaire. This took between 20 and 50 min. Afterward, participants had the opportunity to talk with the investigator in more detail about the purpose of the study and their comparison experiences.

### Materials

The questionnaire that we used in this study, the data tables, and syntaxes can be found on https://osf.io/wchdf/^2^.

#### Self-Report

The self-reports were fictitious but compiled from real internet-blog entries. The supposedly author was a 35-year-old women called Anna, living in Vienna. The poorly adjusted and well-adjusted versions were held as similar as possible. Both self-reports were approximately one-page long. Importantly, the described therapy and prognosis were identical. However, the well-adjusted Anna was much more positive, optimistic, and at ease with the illness. Her self-report read like this (translated from the original German version; see the osf link for the whole questionnaire translated in English):

“…Time flew by and I went back to work quickly. I am optimistic soon to be as productive as before, but I will be more serene. My view on some things in life has changed and I will keep working on this. …”

In the poorly adjusted self-report, Anna described the following:

“…Everything took forever and I had difficulties to get back to work. I am worried I’ll never be as productive as before—my serenity is gone. My view on some things in life has changed and there is nothing I can do about this. …”

#### Perceived Adjustment and Similarity

After having read the self-report, participants rated the adjustment of the standard on a six-point scale (1 = *very poorly*, 6 = *very well*). They also rated their own adjustment in comparison to women with breast cancer in general (1 = *much worse*, 6 = *much better*), their own adjustment in comparison to the self-report standard (1 = *much worse*, 6 = *much better*), and their perceived similarity with the standard (1 = *not at all similar*, 6 = *very similar*).

#### Identification and Contrast

Items adapted from Van der Zee et al. (2000) were used to measure identification and contrast with the patient of the self-report. Van der Zee et al. created these items based on statements collected from 20 audiotaped interviews with women with breast cancer. Moreover, they pretested the items and adjusted them in a pilot study among breast cancer patients.

The items were tailored to the experimental condition because identification and contrast are depicted differently when they refer to an upward standard or to a downward standard. For example, an item measuring identification in the well-adjusted condition read: “When I think of the woman from the self-report, I am glad that my situation could improve.” Conversely, in the poorly adjusted condition identification was phrased: “When I think of the woman from the self-report, I am afraid that my situation will worsen.” Example items for contrast were “When I think of the woman in the self-report, I feel frustrated about my own situation” (well-adjusted standard) and “When I think of the woman from the self-report, I am happy that I am well” (poorly adjusted standard). Participants indicated how much they agreed to two identification and two contrast items on six-point scales (1 = *don’t agree*, 6 = *fully agree*).

#### Focus on Similarities or Differences

The focus on similarities or differences while reading the self-report may carry over to other kinds of comparisons. To assess the propensity to focus on similarities or differences, Mussweiler and Damisch (2008) have created a scale in which participants are asked to judge the similarity of five pairs of every-day objects (e.g., white wine and red wine or a blouse and a dress shirt) on six-point scales (1 = *very different*, 6 = *very similar*). We thus also used this scale to measure our participants’ focus style.

#### Mood

Participants indicated their mood on the single valence item of the Self-Assessment-Manikin with a nine-point answer scale. This mood item consists of a series of figures arranged from smiling on the left (coded as 9) to frowning on the right (coded as 1). This item is used in many studies in which participants’ availability or cognitive capacities make it difficult to employ a more complex measurement (Bynion and Feldner, 2017). The SAM has been validated by Bradley and Lang (1994). Even though the SAM relies on a single item, its authors found that it strongly corelates with longer scales aimed at assessing affective valence. Our participants completed this item twice: before and immediately after reading the self-report.

#### Anxiety and Depression

We used the German version of the Hospital Anxiety and Depression Scale (Herrmann-Lingen et al., 1995) to measure these two dimensions. This scale includes seven items capturing anxiety and seven items capturing depression assessed on four points. An example item for the anxiety subscale is “I feel tense and overexcited” [*most of the time (3)—often (2)—sometimes (1)—not at all (0)*] and for the depression subscale “I can still be as happy today as I used to be [*exactly as then (0)—not quite as much (1)—just a little bit (2)—rarely or not at all (3)*].” We modified the instructions usually associated with this scale: We asked participants to answer according to how they currently felt (instead of “last week” in the original instructions). We changed the wording because we wanted to avoid having participants review what had happened to them in the past week.

#### Self-Esteem and Self-Efficacy

Participants indicated to what extent they agreed with ten items (e.g., “All in all, I am satisfied with myself”; 1 = *not at all true*; 6 = *totally true*) taken from the German version of the Rosenberg self-esteem scale (von Collani and Herzberg, 2003). Moreover, we relied on 10 items to assess their general self-efficacy (e.g., “I have no difficulties to reach my goals and aspirations”; see Schwarzer and Jerusalem, 1995). Participants answered all these items on a six-point scale (1 = *not at all true*, 6 = *totally true*).

## Results

### Perceived Adjustment

As in previous studies on the same topic (e.g., Wood et al., 1985; Stanton et al., 1999), the vast majority (92%) of our participants judged their own adjustment to be superior to patients with breast cancer in general [*M* = 4.89, *SD* = 0.88, *t*(101) = 16.02, *p* < 0.001; testing against the midpoint of the comparative scale].

Participants perceived the patient in the well-adjusted condition as better adjusted (*M* = 5.36, *SD* = 0.96) than the patient in the poorly adjusted condition [*M* = 2.33, *SD* = 1.12, *t*(100) = 14.67, *p* < 0.001, *d* = 2]. However, the well-adjusted patient was not perceived as an upward standard. In both conditions, participants rated themselves to be better adjusted than the women in the self-report, i.e., their answers to this item were on average above the midpoint of the comparative scale at 3.5 [well-adjusted standard: *M* = 4.38, *SD* = 1.05, *t*(49) = 5.94, *p* < 0.001; poorly adjusted standard: *M* = 5.02, *SD* = 1,10, *t*(51) = 10.02, *p* < 0.001].

### Similarity

Participants perceived themselves to be more similar to the well-adjusted (*M* = 4.46, *SD* = 1.72) than to the poorly adjusted standard [*M* = 2.15, *SD* = 1.33, *t*(100) = 7.59, *p* < 0.001, *d* = 1.5].

Several factors may have influenced the way our participants felt similar to the standard. First, the standard that we used in this study was younger (35 years old) than most patients in our sample (on average 63 years old). It could thus be argued that this difference in age prevented our participants to feel similar to the standard and identify with her. Second, more than two-thirds of our participants were currently attending a self-help group or had attended one in the past. This may influence how they related to other women with the same medical condition and, therefore, how they related to the standard. To address these two points, we thus investigated the relation between participants’ age and their assessment of similarity with the standard.

We found that age significantly correlated with the similarity ratings (*r* = −0.31, *p* ≤ 0.001): Over the whole sample, the younger the participants, the more similar they felt to the standard. Did these results mean that only the younger participants of our sample felt similar and identified with the standard? To answer this question, we calculated the mean similarity ratings made by our participants for each age category, i.e., each decade, and for each kind of standard they had been exposed to. These means (see Table 1) show that participants of all age categories reported high levels of similarity with the well-adjusted standard (above 3.8 on a six-point scale) and low levels of similarity with the poorly adjusted standard (below 2.5 on a six-point scale). These results suggest that the young age of the standard did not prevent participants to feel similar to her, when she reported good adjustment.

**TABLE 1:** Mean similarity, identification, and contrast ratings made by participants of different age categories (within brackets are the standard deviation values).

		*N*	Similarity	Identification	Contrast
Poorly	Age < 50	3	1.67 (0.58)	1.67 (0.29)	4.83 (1.26)
adjusted	50 ≤ Age < 60	9	2.44 (1.67)	2.39 (1.69)	4.17 (1.71)
standard	60 ≤ Age < 70	24	2.37 (1.44)	2.13 (1.44)	4.98 (1.01)
	70 ≤ Age < 80	14	1.71 (0.99)	2.00 (1.37)	5.07 (1.07)
	80 ≤ Age < 90	2	2.00 (1.41)	2.50 (0.00)	4.75 (1.77)
Well-	Age < 50	7	5.57 (1.13)	4.71 (1.60)	1.50 (1.32)
adjusted	50 ≤ Age < 60	17	4.82 (1.47)	5.35 (1.09)	1.32 (0.56)
standard	60 ≤ Age < 70	12	3.92 (2.19)	4.17 (1.63)	1.67 (0.94)
	70 ≤ Age < 80	11	3.82 (1.47)	4.82 (1.15)	2.23 (0.96)
	80 ≤ Age < 90	3	3.40 (2.07)	5.50 (0.50)	2.17 (1.61)

*N: number of participants.*

To investigate the effects of age and self-help group attendance on similarity ratings in the context of our study, we ran an ANCOVA with the type of standard as one factor (well vs. poorly adjusted), the attendance to self-help groups as second factor (attending vs. not-attending), age as a covariate, and the similarity ratings as dependent variable. Results indicated a main effect of standard [*F*(1,97) = 44.808, *p* < 0.001, $\eta_{\text{p}}^{2}$ = 0.316]: Participants felt more similar to the well-adjusted (*M* = 4.49, *SE* = 0.234) than to the poorly adjusted standard (*M* = 2.25, *SE* = 0.231). We also found a marginally significant effect of age [*F*(1,97) = 2.827, *p* = 0.096, $\eta_{\text{p}}^{2}$ = 0.028], but the other main effect (attendance to self-help-group) and the interactions were non-significant (all *F*s < 0.551, all *p*s > 0.278).

Taken together these results suggest that our manipulation was effective, and they provide support for our hypotheses. A vast majority of our participants, regardless of their age and attendance to self-help groups, reported high levels of similarity to the well-adjusted standard and low levels of similarity to the poorly adjusted standard.

### Identification and Contrast

**FIGURE 1:**
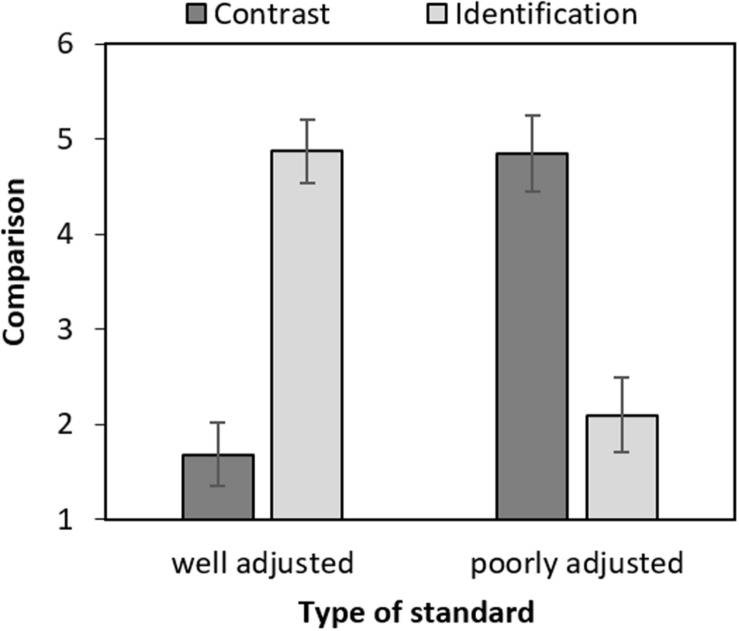
Mean comparison (contrast and identification) by type of standard (poorly vs. well-adjusted). Error bars represent confidence intervals (95%) and were calculated as proposed for within-subject designs by Cousineau and O’Brien (2014).

The internal consistency of both the identification (Cronbach’s α = 0.90; Spearman–Brown’s ρ = 0.90) and contrast (Cronbach’s α = 0.88; Spearman–Brown’s ρ = 0.88) scales was satisfactory^3^. We hypothesized that women with breast cancer perform favorable comparisons, i.e., that they identify with well-adjusted standards and contrast with poorly adjusted ones. This hypothesis was confirmed by a 2 × 2 ANOVA with standard (poorly vs. well-adjusted) as between-subject variable and comparison (identification vs. contrast) as within-subject variable. As depicted in Figure 1, participants reported significantly more identification than contrast for the well-adjusted standard [*F*(1,100) = 143.65, *p* < 0.001, $\eta_{\text{p}}^{2}$ = 0.59], but significantly more contrast than identification for the poorly adjusted standard [*F*(1,100) = 111.03, *p* < 0.001, $\eta_{\text{p}}^{2}$ = 0.52], resulting in a significant interaction [*F*(1,100) = 253.92, *p* < 0.001, $\eta_{\text{p}}^{2}$ = 0.72]. None of the main effects reached significance (*F*s < 1.52, *p*s > 0.221).

To make sure that age and self-help group attendance did not call our results into question, we reran the 2 × 2 ANOVA with standard (poorly vs. well-adjusted) as between-subject variable, comparison (identification vs. contrast) as within-subject variable, and added the two variables (age and self-help group attendance) as covariates. This new analysis led to similar results as the first ANOVA. The interaction between the variables standard and comparison remained significant [*F*(1,97) = 192.591, *p* < 0.001, $\eta_{\text{p}}^{2}$ = 0.665]. *Post hoc* tests using a Tukey correction indicated that participants reported more identification than contrast for the well-adjusted standard [*t*(97) = 11.27, *p*_tuckey_ < 0.001] and more contrast than identification for the poorly adjusted standard [*t*(97) = 10.86, *p*_tuckey_ < 0.001]. We also found a significant main effect of age [*F*(1,97) = 4.297, *p* = 0.041, $\eta_{\text{p}}^{2}$ = 0.042]. Finally, we found a significant interaction between the variables comparison and attendance to self-help groups [*F*(1,97) = 4.359, *p* = 0.039, $\eta_{\text{p}}^{2}$ = 0.043]; however, none of the *post hoc* tests ran to interpret this interaction led to significant differences (all *t*s < 2.437, all *p*s > 0.077). None of the other main effects or interactions reached significance (all *F*s < 1.534, *p*s > 0.219).

### Moderation by Self-Esteem and Self-Efficacy

We analyzed whether self-esteem and self-efficacy moderated the interaction reported in the previous section, i.e., between the variables standard (well vs. poorly adjusted) and comparison process (identification vs. contrast). Both, the self-esteem scale (α = 0.76) and the self-efficacy scale (α = 0.91) were sufficiently internally consistent. Moreover, they correlated with each other (*r* = 0.50, *p* < 0.001).

We ran separate multiple regressions for the contrast and identification scales. We used the macro PROCESS from Hayes (2013) and the bootstrapping method. The predictors were the standard (dummy coded 0 = poorly adjusted, 1 = well-adjusted), the moderator (self-esteem or self-efficacy, centered), and the interaction between both variables. Results indicated a significant Standard × Self-esteem interaction for the contrast scale (*b* = −0.56, *p* = 0.049, 95% CI [−1.12, −0.00]). Simple slope analyses suggested that, in accordance with our hypothesis, the higher participants’ self-esteem the lower their tendency to contrast with the well-adjusted standard. This result was, however, only marginally significant (*b* = −0.36, *p* = 0.066). Moreover, the simple slope analyses for contrasting from the poorly adjusted standard were clearly non-significant (*b* = 0.199, *p* = 0.330). Thus, although significant, the Standard × Self-esteem interaction for the contrast scale should be interpreted with caution. The multiple regressions testing the moderation of self-esteem on the identification scale did not reveal any significant results, nor did the moderation involving self-efficacy (see Supplementary Table S2).

### Focus on Similarities or Differences

The internal consistency of the focus on similarities or differences scale proved to be weak (α = 0.68). Moreover, the scale did not correlate with perceived similarity to either the well-adjusted standard (*r* = 0.20, *p* = 0.176) or the poorly adjusted standard (*r* = 0.08, *p* = 0.575). Therefore, we refrained from performing the analyses we had planned for this scale.

### Mood

**FIGURE 2:**
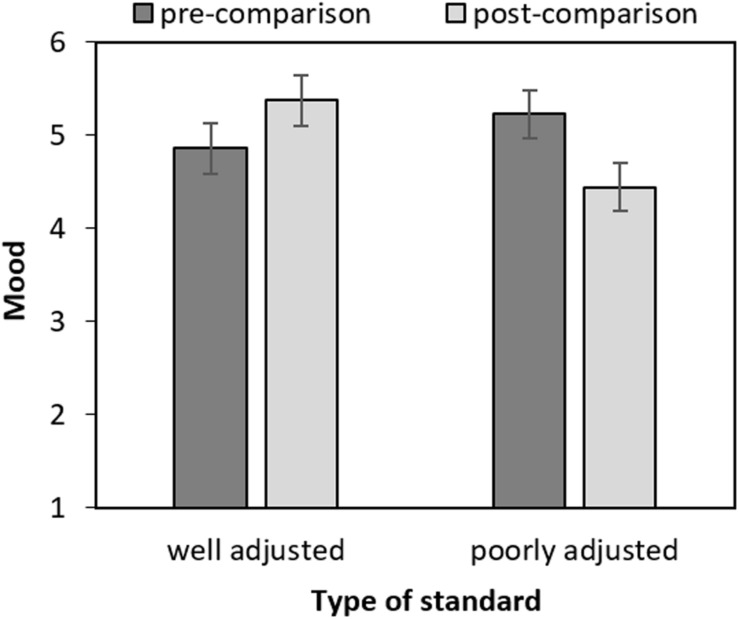
Mood by time (pre- and post-comparison) and type of standard (poorly vs. well-adjusted). Error bars represent confidence intervals (95%) and were calculated as proposed for within-subject designs by Cousineau and O’Brien (2014).

Mood was assessed twice, once before the comparison and once immediately after. A 2 × 2 ANOVA was conducted with standard (poorly adjusted vs. well-adjusted) as between-subject factor and time (pre-comparison vs. post-comparison) as within-subject factor. The interaction was significant [*F*(1,100) = 23.32, *p* < 0.001, $\eta_{\text{p}}^{2}$ = 0.19], but none of the main effects were (*F*s < 1.01, *p*s > 0.317). As depicted in Figure 2, participants’ mood increased after they had read the well-adjusted standard report [*F*(1,100) = 7.22, *p* = 0.008, $\eta_{\text{p}}^{2}$ = 0.07] and decreased after the poorly adjusted standard report [*F*(1,100) = 17.27, *p* < 0.001, $\eta_{\text{p}}^{2}$ = 0.15]. Mood did not significantly differ between condition before the self-report reading [*F*(1,100) = 1.56, *p* = 0.215, $\eta_{\text{p}}^{2}$ = 0.015], but it did afterward [*F*(1,100) = 8.22, *p* = 0.005, $\eta_{\text{p}}^{2}$ = 0.08]. Thus, participants’ mood assimilated to the mood of the standard.

**FIGURE 3:**
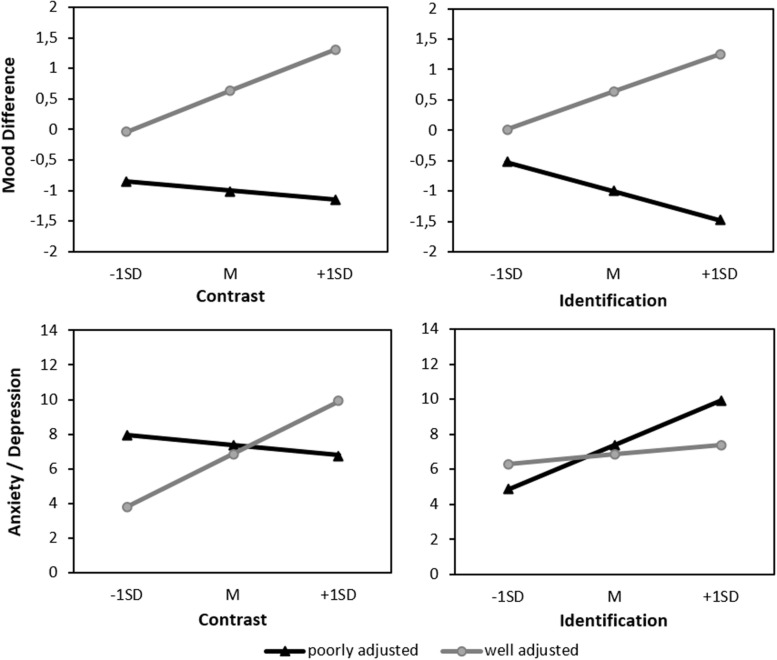
Upper panel: Predicted mood difference (“mood after”—“mood before reading the self-report”). Lower panel: Anxiety/depression by standard (well or poorly adjusted) and contrast/identification.

We hypothesized that the affective reaction to the comparison depends on the type of comparison process. For example, the more participants identify with the poorly adjusted standard, the more their mood shall decrease. To test such moderation effects, we regressed mood differences on the Standard, Contrast, Identification variables, and the interactions of Standard × Contrast and Standard × Identification. We found a significant Standard × Identification interaction (see Table 2 and Figure 3 upper panel on the right). Simple slopes indicated that the more participants identified with the well-adjusted standard the stronger the increase in their moods (*b* = 0.32, *p* = 0.026). The slope for identification with the poorly adjusted standard displayed the opposite effect but was only marginally significant (*b* = −0.24, *p* = 0.078). Although these results are in line with our hypotheses, they should be interpreted with caution. Indeed, we observed a similar pattern for the contrast scale although we had predicted the opposite (see Figure 3, upper panel on the left). The Standard × Contrast interaction was not significant (see Table 2) but means suggest that, contrary to our hypothesis, the more participants contrasted from the standard the more similar to the standard’s their mood became.

**TABLE 2:** Moderation analyses predicting mood difference and anxiety/depression.

	*B*	*SE*_b_	*p*
**Mood differences**			
Constant	−1.00 [−1.65, −0.36]	0.33	0.003
Standard	1.63 [0.59, 2.68]	0.53	0.002
Contrast (centered)	−0.08 [−0.39, 0.24]	0.16	0.632
Identification (centered)	−0.24 [−0.52, 0.03]	0.14	0.078
Standard × Contrast	0.43 [−0.08, 0.93]	0.25	0.095
Standard × Identification	0.56 [0.17, 0.95]	0.20	0.005
*R*^2^	0.28		
**Anxiety/depression**			
Constant	7.37 [6.07, 8.68]	0.66	<0.001
Standard	−0.51 [−2.62, 1.60]	1.06	0.631
Contrast (centered)	−0.31 [−0.95, 0.33]	0.32	0.340
Identification (centered)	1.31 [0.76, 1.86]	0.28	<0.001
Standard × Contrast	1.90 [0.89, 2.91]	0.51	<0.001
Standard × Identification	−1.03 [−1.82, −0.24]	0.40	0.011
*R*^2^	0.32		

*Standard is coded 0 = poorly adjusted and 1 = well-adjusted. Square brackets contain 95% confidence intervals for b.*

### Anxiety and Depression

The anxiety subscale (α = 0.79) as well as the depression subscale (α = 0.82) both showed sufficient internal consistency and correlated highly with each other (*r* = 0.65, *p* < 0.001). For ease of interpretation and brevity, we averaged the sum-score of the two subscales^4^.

The anxiety/depression score was regressed on Standard, Identification, Contrast, and the interactions Standard × Identification and Standard × Contrast. Both interactions significantly predicted the anxiety/depression score (see Table 2) indicating a moderation of the effect of the standard depending on the type of comparison process (see Figure 3, lower panel). Simple slope analyses specified that the more participants contrasted from the well-adjusted standard (*b* = 1.59, *p* ≤ 0.001) and the more they identified with the poorly adjusted standard (*b* = 1.31, *p* < 0.001), the more anxiety and depression they reported. The remaining slopes were not significant (identification with well-adjusted standard: *b* = 0.28, *p* = 0.331; contrast to poorly adjusted standard: *b* = −0.31, *p* = 0.340).

## Discussion

This study was designed to examine the reactions of patients with breast cancer after reading the report of either a well or poorly adjusted fellow patient. Consistent with our hypotheses, we found that when faced with a well-adjusted patient, participants reported more identification than contrast, whereas when faced with a poorly adjusted patient, the opposite occurred. This indicates that the patients were able to adjust their comparison to the standard and, thereby, accomplish favorable comparison processes. Moreover, we found that the type of comparison predicted self-assessment of anxiety and depression after the comparison. The less participants contrasted with the well-adjusted standard and identified with the poorly adjusted standard, the less anxiety and depression they reported. These results suggest that avoiding unfavorable comparison processes is especially beneficial, even more so than engaging in favorable ones. In the context of our study, these results may be related to the fact the participants showed a high level of favorable comparison processes in general.

The mood measure revealed a strong assimilation effect. Immediately after reading that another breast cancer patient had adjusted well, was optimistic and spirited, the women themselves felt happier than after reading the report of a poorly adjusted woman. This effect was more pronounced the more the women identified with the standard. However, there was also a tendency indicating that more contrastive comparison was associated with more mood assimilation. This tendency stands in sharp contrast to the expected consequence of such a comparison (Van der Zee et al., 2000; Mussweiler, 2003). Apparently, the more our participants compared with the standard, regardless whether they later described this comparison process as contrast or identification, the more they showed an immediate, assimilative mood reaction. There are several possible explanations for this result. First, some people argue that assimilation is the primary or more natural mechanism in social comparison (Mussweiler, 2003). Therefore, the immediate affective reaction might be guided by this mechanism and only people who do not compare at all remain unaffected by the standard. Second, assimilation is fostered by similarity (Mussweiler, 2003). In our study, there was an important and obvious similarity between the participants and the standard: both were women who have survived breast cancer. This similarity could explain why participants assimilated their mood to the positive or negative tone of the other patients’ self-report. Finally, one could interpret these results as an expression of sympathy for the standard rather than the consequence of a comparison process. Participants may indeed feel saddened by the report of the poorly adjusted standard and elated by the report of a well-adjusted standard whether they identified with her or not. Future research should investigate the mechanisms of this effect.

When planning this study, we speculated that patients with high self-esteem or high self-efficacy would be more likely to engage in favorable comparison strategies and might therefore profit more from these comparisons. Our results did not support this hypothesis. Except for one interaction (i.e., the moderation of self-esteem on the contrast scale), self-esteem and self-efficacy did not moderate the comparison process itself. This suggests that, contrary to our predictions, people with high self-esteem or high self-efficacy may not profit more from these comparisons.

Even though it is a strength of the present study that the type of comparison was assessed, it is also a weakness. This method allowed us to detect that patients with breast cancer react with favorable comparison processes toward fellow patients, but it limits the interpretation of this effect on affective reactions. Due to the correlative design, it remains unclear whether women who contrast more from a well-adjusted patient feel more anxiety and depression, or whether women who feel more anxiety and depression contrast more from well-adjusted patients. To disentangle both hypotheses, one would need to experimentally manipulate identification and contrast.

In addition, it is important to mention that the items to assess identification and contrast in this study already incorporated an affective component (e.g., “If I think about the women in the text, I am anxious that my situation will get worse.”). These items were modeled after those of Van der Zee et al. (2000) and aimed to differentiate between favorable and unfavorable comparison processes. However, it would be interesting to measure identification and contrast without this affective component and, for example, assess participants focus on or thinking about similarities between themselves and the other person while doing the comparison (for similar methods see Petersen et al., 2012; Arigo et al., 2015). In our study, we included the similarity and dissimilarity focus measure for this purpose (Mussweiler and Damisch, 2008). Unfortunately, this measurement turned out to be invalid. More reliable measurements need to be developed to deepen our understanding of comparison processes.

We also want to point out further limitations concerning the generalizability of the results based on the manipulation and sample. First, we manipulated the level of adjustment in the self-report but did not vary information about the prognosis. We used this manipulation because prior research indicates that patients react differently toward these two types of information (Stanton et al., 1999). Moreover, one may argue that in daily encounters the other person’s adjustment is more easily detectable than her prognosis (see Graves et al., 2005) and that comparisons with this kind of information is more likely. However, one would expect that knowing the standard’s prognosis or reading a self-report that focuses on other aspects of the cancer experience triggers different comparison process and especially different affective reactions (see Buunk et al., 2009). In general, it would be valuable to explore further, whether the self-serving effects that we observed in this study can be replicated with different types of standards and patients.

Another potential limitation of our study relates to the fact that we introduced the women from the self-report to be 35 years old. This age is much younger than the age of the women in our sample (*M*_age_ = 63). As outlined above, comparisons are easily influenced by perceived similarity to the comparison standard and people tend to prefer similar others as comparison standard (Goethals and Darley, 1977). In line with this hypothesis, our results indicated that the younger the participants the more similar they felt to the standard, and the less they contrasted from her. This result raised the question of whether the older part of our sample perceived any similarity between them and the standard and could identify with her. In support of this notion, we found that participants of all age categories reported high levels of similarity and identification with the well-adjusted standard and low levels of similarity and identification with the poorly adjusted standard (see Table 1). Moreover, we reran our main analyses with age as a covariate and found that the effect of the standard remained highly significant. Altogether, these results suggest that, indeed, our younger participants felt more similar to the standard than our older ones; however, all of them identified strongly with the standard when she reported a good adjustment. These results also suggest that the standard’s adjustment (poorly adjusted vs. well-adjusted) plays a much bigger role than age in the extent to which patients identify with her.

When patients with breast cancer encounter another patient, they can rely on many personal characteristics to identify similarities or differences. Age is of them, but our data suggest that the standard’s level of adjustment is more determinant. The fact that age did not play a major role in our participants’ ratings of similarity and identification is in line with the results obtained by Wood et al. (1985). They examined the narrations of women with breast cancer and found that patients with breast cancer compare themselves to other patients with cancer but other than suffering of the same disease, the actual similarity with the standard did not seem to play a big role. These results, therefore, not only reveal that our manipulation was efficient, but they also support our main hypothesis that women with breast cancer flexibly adjust their comparison processes to their advantage.

Besides these limitations, this study clearly indicates that breast cancer patients’ affective reaction toward other patients do not only depend on the type of standard they are exposed to—i.e., whether the other patient is doing well or poorly—but is also related to the type of comparison processes they engage in. Social comparisons can induce both positive and negative feelings. It was thus reassuring to see that in our sample, participants predominantly engaged in favorable comparison processes. However, it might be wise to prepare patients who are newly diagnosed with breast cancer for these unavoidable comparisons and to provide further guidance. Being in contact with fellow patients, sharing experiences, and getting social support can have a positive impact on patients’ well-being (Gray et al., 1997; Manning and Dickens, 2007; Stang and Mittelmark, 2008) and no woman should isolate herself out of fear of unfavorable social comparisons.

## Data Availability Statement

All datasets generated for this study are freely available in the OSF (https://osf.io/wchdf/).

## Ethics Statement

The studies involving human participants were reviewed and approved by the Ethics Commission of the University of Graz (Austria). The patients/participants provided their written informed consent to participate in this study.

## Author Contributions

KC, RI, and HI conceived the project hypotheses and designed the experimental material. RI and HI collected the data. RI, HI, and GK conducted the analyses. KC, GK, RI, and HI contributed to the interpretation of the results and the writing of the manuscript.

## Conflict of Interest

The authors declare that the research was conducted in the absence of any commercial or financial relationships that could be construed as a potential conflict of interest.

## Footnotes

1 We did not run a power analysis before collecting and analyzing the data. However, *post hoc* calculations of power reveal that assuming a small effect size (*f* = 0.3), our sample of 102 participants had a power = 0.851 to detect a significant difference between the comparison processes of our two groups of participants (i.e., based on the two-way ANOVA reported in section “Identification and Contrast”).

2 To protect the anonymity of our participants, we removed some of their demographical variables from the data table.

3 We report both the Cronbach’s alpha and the Spearman–Brown’s coefficient because the combination of these two coefficients seems to lead to the most robust assessment of reliability of two-item scales (see Eisinga et al., 2013).

4 Analyses of the anxiety and depression scales separately reveal similar results are those found for the average score of both scales.
