# Supplementary material for: Affective Consequences of Social Comparisons by Women With Breast Cancer: An Experiment
Source: Front Psychol. 2020 Jun 11;11:1234. doi: 10.3389/fpsyg.2020.01234 (PMC7300312; doi:10.3389/fpsyg.2020.01234)
Supplement: Supplementary file 1 [file Table_1.DOCX]

# Supplemental Material

Table 1

*Description of the Sample*

|  | *Frequencies* | | | | | | | | | | | | | | | | | | |
| --- | --- | --- | --- | --- | --- | --- | --- | --- | --- | --- | --- | --- | --- | --- | --- | --- | --- | --- | --- |
| Age of participants | *30-39*  2 | | *40-49*  8 | | | | *50-59*  26 | | | | *60-69*  36 | | | *70-79*  25 | | | | *80-89*  5 | |
| Family status | *Single*  4 | | | *Married*  64 | | | | | *Partnership*  5 | | | *Widowed*  20 | | | | *Divorced*  9 | | | |
| Employment | *Labourer*  2 | | *Employee*  20 | | | | *Self-employed*  6 | | | | *Retired*  63 | | *Unemployed*  4 | | | | *Occupational disable*  7 | | |
| Education | *Compulsory School*  24 | | *Apprentice-ship*  39 | | | | *Secondary School*  17 | | | | *High School Diploma*  3 | | | *Higher Education*  18 | | | | *Missing*  1 | |
| First Cancer Diagnosis | *1980-84*  1 | *1985-89*  6 | | | | *1990-94*  9 | | *1995-99*  10 | | | *2000-04*  13 | | *2005-09*  24 | | *2010-14*  24 | | | | *2015-19*  15 |
| Number of Diagnosis | *One*  85 | | | | *Two*  12 | | | | | *Three*  5 | | |  | |  | | | |  |
| Operations | *None*  5 | | | | *One Breast*  87 | | | | | *Both Breasts*  10 | | |  | |  | | | |  |
| Currently in therapy? | *Yes*  25 | | | | *No*  77 | | | | |  | | |  | |  | | | |  |
| Member of self-help group? | *Yes*  66 | | | | *Not at the moment*  3 | | | | | *Never*  33 | | |  | |  | | | |  |

*Note.* Data were collected in 2015.

Table 2

*Moderation Analyses Predicting Contrast from and Identification with the Standard (i.e. the patient from the self-report)*

|  | Contrast | | |  | Identification | | |
| --- | --- | --- | --- | --- | --- | --- | --- |
|  | *b* | *SE_b_* | *p* |  | *b* | *SE_b_* | *p* |
| Standard and  self-esteem |  |  |  |  |  |  |  |
| Constant | 4.85  [4.55, 5.15] | 0.15 | < .001 |  | 2.09  [1.71, 2.46] | 0.19 | < .001 |
| Standard | -3.16  [-3.59, -2.74] | 0.21 | < .001 |  | 2.78  [2.25, 3.32] | 0.27 | < .001 |
| Self-esteem (centred) | 0.20  [-0.20, 0.60] | 0.20 | .330 |  | -0.44  [-0.94, 0.07] | 0.26 | .090 |
| Standard x  Self-esteem | -0.56  [-1.12, -0.00] | 0.28 | .049 |  | 0.36  [-0.34, 1.07] | 0.36 | .310 |
| *R*² | .70 |  |  |  | .53 |  |  |
| Standard and  self-efficacy |  |  |  |  |  |  |  |
| Constant | 4.84  [4.54, 5.14] | 0.15 | < .001 |  | 2.10  [1.73, 2.48] | 0.19 | < .001 |
| Standard | -3.16  [-3,59, -2,73] | 0.22 | < .001 |  | 2.77  [2.23, 3.31] | 0.27 | < .001 |
| Self-efficacy (centred) | 0.17  [-0.16, 0.50] | 0.17 | .307 |  | -0.26  [-0.67, 0.15] | 0.21 | .217 |
| Standard x  Self-efficacy | -0.21  [-0.67, 0.25] | 0.23 | .360 |  | 0.43  [-0.15, 1,00] | 0.29 | .143 |
| *R*² | .69 |  |  |  | .52 |  |  |
| *Note.* Standard is coded 0 = poorly-adjusted and 1 = well-adjusted. Square brackets contain confidence intervals for *b*. | | | | | | | |
